# A transdisciplinary approach to the initial validation of a single cell protein as an alternative protein source for use in aquafeeds

**DOI:** 10.7717/peerj.3170

**Published:** 2017-04-11

**Authors:** Michael Tlusty, Andrew Rhyne, Joseph T. Szczebak, Bradford Bourque, Jennifer L. Bowen, Gary Burr, Christopher J. Marx, Lawrence Feinberg

**Affiliations:** 1 Anderson Cabot Center for Ocean Life at the New England Aquarium, New England Aquarium, Boston, MA, United States; 2 School for the Environment, University of Massachusetts Boston, Boston, MA, USA; 3 Department of Arts and Sciences, Roger Williams University, Bristol, RI, United States; 4 Department of Biology and Marine Biology, Roger Williams University, Bristol, Rhode Island, United States; 5 Northeastern University, Nahant, MA, United States; 6 National Cold Water Marine Aquaculture Center, USDA ARS, Franklin, ME, United States; 7 KnipBio Inc., Lowell, MA, United States

**Keywords:** Biotechnology, Aquaculture, Single cell protein, Shrimp, Salmon, Methylotrophs, Alternate protein, Food security, Microbiome, Smallmouth grunt

## Abstract

The human population is growing and, globally, we must meet the challenge of increased protein needs required to feed this population. Single cell proteins (SCP), when coupled to aquaculture production, offer a means to ensure future protein needs can be met without direct competition with food for people. To demonstrate a given type of SCP has potential as a protein source for use in aquaculture feed, a number of steps need to be validated including demonstrating that the SCP is accepted by the species in question, leads to equivalent survival and growth, does not result in illness or other maladies, is palatable to the consumer, is cost effective to produce and can easily be incorporated into diets using existing technology. Here we examine white shrimp (*Litopenaeus vannamei*) growth and consumer taste preference, smallmouth grunt (*Haemulon chrysargyreum*) growth, survival, health and gut microbiota, and Atlantic salmon (*Salmo salar*) digestibility when fed diets that substitute the bacterium *Methylobacterium extorquens* at a level of 30% (grunts), 100% (shrimp), or 55% (salmon) of the fishmeal in a compound feed*.* In each of these tests, animals performed equivalently when fed diets containing *M. extorquens* as when fed a standard aquaculture diet. This transdisciplinary approach is a first validation of this bacterium as a potential SCP protein substitute in aquafeeds. Given the ease to produce this SCP through an aerobic fermentation process, the broad applicability for use in aquaculture indicates the promise of *M. extorquens* in leading toward greater food security in the future.

## Introduction

Aquaculture is the fastest growing source of animal protein for humans. However, as this industry continues to develop, several inherent challenges will arise. Foremost is the need for increased production of suitable and sustainable feeds. Aquaculture has long been criticized for “using fish protein to make fish protein” (Naylor et al., 2009). This “fishmeal trap” (New & Wijkström, 2002) caused the industry to prioritize improving feed conversion ratios and finding alternative protein sources (Naylor et al., 2009). While soy is the most common terrestrial plant protein used in fishmeal substitution to date, many environmental concerns surround the land-use and fertilizer run-off requirements associated with soy production. Additionally, palatability and anti-nutritional factors, as well as unintended biological consequences (*e.g.*, gastroenteritis in salmon, Romarheim et al., 2011), limit the immediate and broad application of unmodified soy and other plant proteins.

Alternatively, single cell proteins (SCP), mainly yeast, algae, and bacteria, show much promise for aquaculture (Naylor et al., 2009). SCP has historical roots in Germany when, during the First World War, approximately 50% of imported protein was offset by yeast (Suman et al., 2015). Today, spent yeast cells from corn ethanol fermentation processes are commonly blended with dried distiller’s grains and solubles in terrestrial animal feeds (Kim & Dale, 2004). However, the high fiber content of this blend limits its use in aquaculture (Gatlin et al., 2007). Similarly, algae are grown commercially in ponds or bioreactors for use in food, cosmetics, oil and nutritional supplements. To date, the large scale application of algae as an alternative protein source is limited by high production costs and technical challenges (Spolaore et al., 2006).

Bacterial biomass, while currently the least developed SCP, has potentially great applicability as a protein replacement for aquaculture. Here we test the applicability of *Methylobacterium extorquens*, an abundant leaf symbiont that can grow rapidly and to high densities on the non-food, single-carbon (C_1_) feedstock methanol (Schrader et al., 2008). Given the large production levels of natural gas-derived methanol, this has driven a new wave of research into C_1_ biotechnology, much of which has been with *M. extorquens* (Ochsner et al., 2015). *M. extorquens* has attracted this attention as the premier model organism for growth on C_1_ compounds because of its relative metabolic versatility, the large suite of genetic tools developed for it, and the availability of full genome sequences for multiple strains (Vuilleumier et al., 2009; Marx et al., 2012). Being produced through a fermentation process, this SCP is immune to seasonality or other undue climate influences (e.g., extreme temperatures, droughts, floods). One particular endogenous trait that provides advantage is that *M. extorquens* contains a suite of naturally occurring anti-oxidant carotenoid compounds that have been associated with both imparting color and enhancing immunity (Osawa et al., 2015; Van Dien et al., 2003). Carotenoid compounds, such as astaxanthin and canthaxanthin, are commonly added to aquaculture feeds to provide aquacultured product with the color of wild counterparts (Tlusty & Hyland, 2005). Carotenoid compounds often represent one of the most expensive ingredients in feed. Also, some carotenoids are precursors of vitamin A and many have antioxidant properties important to immune systems. The principal method for the manufacturing of pigments to serve the aquaculture industry is often by chemical synthesis, which is restricted for use in certain jurisdictions (e.g., European Union). These natural traits of *M. extorquens* offer a uniquely powerful opportunity to rapidly advance and tailor SCP for specific nutritional benefits.

Before the full potential of SCP for aquaculture can be realized, a number of interdisciplinary first principles must be established (Engle, 2016; Rhodes, Johnson & Myers, 2016). These include demonstrating that diets containing SCP (1) are accepted by the species in question, (2) result in equivalent survival and growth as individuals raised with traditional feeds, (3) do not cause illness or other maladies, (4) result in an organoleptically suitable product, and (5) are cost effective to manufacture and feed.

Here, we describe the production and use of KnipBio Meal (KBM), a novel high-yielding platform biocatalyst using *Methylobacterium extorquens,* as an effective protein source for aquafeeds. We tested this SCP as a potential feed item in two species of commercial aquaculture value (Pacific white shrimp, *Litopenaeus vannamei,* and, Atlantic salmon, *Salmo salar*) and one of ornamental aquaculture value (smallmouth grunt, *Haemulon chrysargyreum,* Tlusty et al., 2017). Specifically, we conducted feeding trials using Pacific white shrimp and smallmouth grunt to determine the effect of KBM (up to 30% or 100% fishmeal inclusion rates, depending on species) on animal growth, health, and survival. Additionally, we conducted a trial using Atlantic salmon to determine the digestibility of the KBM compared to a commercially available reference diet. Together, these data represent the initial trials for the feasibility of KBM as a suitable SCP for use in aquafeeds.

## Methods

### Single cell protein biomass and feed pellet formulation

*Methylobacterium extorquens* (strain KB203) was produced via standard aerobic fermentation processes (Bélanger et al., 2004) and de-watered to form a flour referred to as KnipBio Meal (KBM). KB203 was incubated at 30 °C at 200 RPM on CHOI4 liquid medium (Supplemental Table 1) with 0.5% methanol for 24 hr in 50 mL liquid medium (in a 250 mL baffled flask). To determine purity, the suspension was streaked onto tryptic soy agar and incubated at 30 °C for 96 hr. Only colonies of a single morphology were considered pure and fit for further use in scale-up.

CHOI4-defined medium and trace metals stock solution recipes were used for growing *M. extorquens* to high cell densities. The trace metals solution was prepared separately as a concentrate and autoclaved for 30 min at 121 °C. A 30 mL trace metals solution was added to the medium before sterilization. Although precipitation is often observed in these solutions, they have been used repeatedly with success for growing *M. extorquens* to high cell densities (Bélanger et al., 2004).

A 20 L fermenter (equipped with two Rushton-type impellers; Chemap, Uster, Switzerland) was used for growing the inoculum for the main fermenter. Two pH probes (Mettler, Toledo), two pO_2_ probes (Ingold) and one methanol probe (volatile organic compound (VOC) probe; NRC, Montreal, Canada) were prepared and fit into the 20L fermenter before sterilization. Ten liters of CHOI4 medium was prepared and sterilized in the fermenter for 45 min. After cooling to room temperature, a two-point calibration was conducted on the methanol probe by aseptically adding two defined volumes of methanol to reach a final concentration of 0.18% in the fermenter (2 × 9 mL). Sterile ammonium hydroxide and methanol were connected to the fermenter to control pH (at 7.0) and methanol concentration. The pO_2_ and methanol probes were calibrated under standard minimal positive pressure (0.05 bar). Final fermentation occurred in a 1500 L fermenter (equipped with three Rushton-type impellers and a mechanical foam breaker; Chemap, Uster, Switzerland) with similar specifications to seed fermentation, prepared with two pH probes (Mettler Toledo, Columbus, OH, USA), two pO_2_ probes (Ingold and Hamilton, Reno, NV, USA) and two methanol probes (NRC, Montreal, Canada). The probes were prepared and fit into the fermenter before sterilization. 

For bench top fermentations, biomass was collected through centrifugation (Beckman-Coulter, Indianapolis, IN, USA) and freeze dried for 48 hr at −80 °C, and moisture content was verified to be below 10% (New Jersey Feed Labs, Ewing Township, NJ, USA). At larger scale, the biomass was harvested by cooling to 20 °C and pressure was increased to 0.8 bar before feeding to the BTPX 205 disc stack centrifuge (Alfa-Laval, Lund, Sweden) at 100L/hr (discharged every 2 min). The slurry was sent directly into 50 L polypropylene carboys and stored at 4 °C until further treatment. After approximately 24 hr, the slurry was spray dried at 182 °C/70 °C (inlet/outlet).

### Diet formulation

Experimental diets used for all animal trials were produced using commercial manufacturing methods. Specifically, for both Pacific white shrimp (*Litopenaeus vannamei,* Table 1) and Atlantic salmon (*Salmo salar,* Table 2) trials, ingredients were ground to a particle size of <200 µm using an air-swept pulverizer (Model 18H; Jacobsen, Minneapolis, MN, USA). The diets were processed using a twin-screw cooking extruder (DNDL-44, Buhler AG, Uzwil, Switzerland) with a 25 sec exposure to 127 °C in the extruder barrel (average across five sections). Pellets were dried with a pulse bed drier (Buhler AG, Uzwil, Switzerland) for 20 min at 102 °C with a 10 min cooling period, resulting in final moisture levels less than 10%. All oil was top-coated after the pellets were cooled using a vacuum-coater (AJ Mixing, Ontario, CA, USA). Yttrium oxide was added to the reference diet at 0.1% of dry weight to serve as an inert, indigestible markerand was diluted to 0.07% when the test ingredients were added. Diets were stored in polypropylene plastic bags at room temperature until fed. All diets were fed within four months of manufacture.

**Table 1:** Experimental feeds. Composition of three experimental feeds used to test the efficacy of KnipBio single cell protein (KnipBio meal; KBM) as a fishmeal substitute using Pacific white shrimp (*L. vannamei*), where SHR-C, **SHR**imp **C**ontrol feed (modelled after Jobling, 2012) and SHR-KL and SHR-KH are control feed with fishmeal replaced with KBM; KL, **K**nipBio meal **L**ow (50% replacement) and KH, **K**nipBio meal **H**igh (100% replacement).

Ingredient	Composition (g kg^−1^ as fed)		
	Control	50% KBM	100% KBM
Menhaden fish meal^1^	120.0	60.0	0.0
KnipBio meal^2^	0.0	63.0	126.0
Soybean meal^3^	380.0	380.0	380.0
Menhaden fish oil^4^	30.7	37.1	43.5
Corn starch^5^	34.8	17.4	0.0
Whole wheat^6^	340.0	340.0	340.0
Trace mineral premix^7^	5.0	5.0	5.0
Vitamin premix^8^	18.0	18.0	18.0
Choline chlorine^9^	2.0	2.0	2.0
Stay C^10^	1.0	1.0	1.0
CaP-diebasic^11^	20.0	28.0	36.0
Lecithin^12^	10.0	10.0	10.0
Cholesterol^13^	0.5	0.5	0.5
Empareal 75 CGM^14^	38.0	38.0	38.0

**Table 2:** Salmon diet. Composition of two experimental feeds used to test the digestibility of KnipBio single cell protein (KnipBio meal; KBM) as a fishmeal substitute using Atlantic salmon (*S. salmar*), where **SAL-C**, **SAL**mon **C**ontrol diet (*modelled after Gaylord et al., 2009*).

Ingredient	Composition (g kg^−1^ as fed)
	Sal-C
Squid meal	260.0
Soy protein concentrate	171.4
Corn gluten meal	83.4
Soybean meal	43.0
Wheat flour	283.3
Taurine	5.0
Menhaden fish oil	133.9
Vitamin premix, ARS 702	10.0
Choline chlorine	6.0
Vitamin C	2.0
Yttrium oxide	1.0
Trace mineral premix	1.0

For smallmouth grunt (*Haemulon chrysargyreum*) trials (Table 3), all dry ingredients were mixed using a feed mixer (Model KSMS, Kitchen Aid Inc.; St. Joseph, Michigan, USA). Pollock liver oil and lecithin were added to the mixture followed by about 45% distilled water to aid the pelleting process via meat chopper (Royal, Tokyo, Japan, type 22VR-1500). After pelleting, all diets were dried at 70 °C in a constant temperature oven (DK 400; Yamato Scientific Co., Ltd., Tokyo, Japan). The dried pellets were steamed at 100 °C for 1 min in a cylindrical steamer to improve water stability. Pellets were stored in plastics bags at 30 °C until used. All diets were fed within four months of manufacture.

**Table 3:** Grunt experiment. Composition of four experimental feeds used to test the efficacy of KnipBio single cell protein (KnipBio meal; KBM) as a fishmeal substitute using smallmouth grunt (*H. chrysargyreum*), where GRU-C1, **GRU**nt **C**ontrol feed (modelled after Alam et al., 2012; Alam, Watanabe & Carroll, 2008; Alam et al., 2009), GRU-C2, GRU-C1 with 80 ppm carotenoid addition, and GRU-KL and GRU-KH are control feed with fishmeal replaced with KBM; KL, **K**nipBio meal **L**ow (10% replacement) and KH, **K**nipBio meal **H**igh (50% replacement).

Ingredient	Composition (g kg^−1^ as fed)			
	GRU-C1	GRU-C2^*^	GRU-KL	GRU-KH
Menhaden fish meal	500.0	500.0	470.0	350.0
KnipBio meal	0.0	0.0	50.0	250.0
Squid meal	100.0	100.0	100.0	100.0
Soy bean meal	100.0	100.0	100.0	100.0
Wheat starch	70.0	70.0	60.0	30.0
Wheat gluten	50.0	50.0	50.0	50.0
Menhaden fish oil	50.0	50.0	55.0	60.0
Soybean lecithin	10.0	10.0	10.0	10.0
Vitamin premix	20.0	20.0	20.0	20.0
Trace mineral premix	20.0	20.0	20.0	20.0
Alpha-cellulose	70.0	66.0	55.0	0.0
Astaxanthin	0.0	4.0	0.0	0.0
Methionine	5.0	5.0	5.0	5.0
Lysine	5.0	5.0	5.0	5.0

**Notes.**

* Diet GRU-C2 was identical to GRU-C1 with an added 80 ppm carotenoid.

### The effect of KBM on growth, survival, and feed efficiency of the Pacific white shrimp

Hatchery-raised Pacific white shrimp (*Litopenaeus vannamei*) were acquired from SKY8 Shrimp Farm, LLC (Stoughton, MA, USA) and stocked at 60 shrimp/tank (shrimp average weight was 4.52 ± 0.21 g (1.S.D.)) into twelve 110 L glass aquaria (0.228 m^3^) comprising a 1,675 L single clear water recirculating saltwater aquaculture system with mechanical and biological filtration. Experimental systems were maintained at 27.5–28.5 °C, and 29.5–32.5 ppt salinity, and ammonia, nitrite, and nitrate were maintained at ≤0.25, ≤0.25, and ≤80.00 ppm, respectively. This density was greater for the initial stocking given that destructive sampling would take place. The final stocking density is on par with that practices by intensive land-based systems. Animal care and procedures used in this trial were approved by Roger Williams University Animal Care and Use Committee (IACUC protocol R-13-12-20).

To determine the effect of KBM on shrimp growth and survival, three diets of varying KBM inclusion were formulated (Table 1). Each of the 12 experimental tanks was randomly assigned one of the three diets, totaling four replicates per treatment. Each tank was fed to apparent satiation four times/day (800, 1100, 1400, 1600 hours). Uneaten food from the previous feed, as well as any excrement or molts, was manually siphoned from each tank prior to the next feeding. Water chemistry was tested and corrected daily throughout the duration of the experiment.

The gross wet weight (g) of all shrimp per tank was measured at days 0, 60, and 150. All shrimp were measured on day 0 (*n* = 60), and day 60 (*n* = 45–55, depending on survivorship). At day 60, 20 shrimp from each tank (*n* = 80 per treatment) were randomly selected and returned to their original tank (a density of 87 m^−2^) and maintained according to the above experimental design for an additional 90 days, as which point each individuals was enumerated for wet weight (g) and carapace length (mm). The shrimp not selected for the second 90-day trial (*n* = 25–35, depending on tank) were euthanized, placed on ice, and wet weight (g), and carapace length (mm) were measured for each individual.

A blind taste test was conducted immediately following the conclusion of the second 90-day trial. After final data were collected, the shrimp were grouped by treatment, placed on ice, and transported to JR Bean Saloon in Bristol, RI, USA for preparation for human consumption. Shrimp were aggregated by treatment and were subsequently shelled, de-veined, boiled, and immediately served to 39 test participants. Diet treatment identity was concealed from test participants using colored plastic forks, one color per treatment. To randomize the order of shrimp consumption per participant, each fork was randomly labeled with a number (1, 2, or 3), indicating the order in which each participant should consume his/her shrimps. Upon completing the tasting, each participant ranked the three shrimp based on overall taste from 1–3 by placing the corresponding fork from each shrimp into one of three buckets. Participants were given the option to vote for a “tie”, however, these votes only accounted for 5% of total votes, and were not included in the analysis.

### The effect of KBM on smallmouth grunt growth, proximate composition, and gut microbiome

Hatchery-raised smallmouth grunts (*Haemulon chrysargyreum*; *N* = 120; 1.37 ± 0.27 g wet wt) were stocked at 10 fish/tank into twelve 110 L glass aquaria (0.113 m^3^) comprising a 1,675 L recirculating saltwater aquaculture system with mechanical and biological filtration. Each of the 12 experimental tanks was randomly assigned one of four experimental diets, totaling three replicates per treatment (Table 2). Each tank was fed until apparent satiation four times/day (800, 1100, 1400, 1600 hours). Uneaten food from the previous feed, as well as any excrement, was manually siphoned from each tank prior to the next feeding. Water chemistry was tested and corrected daily throughout the duration of the experiment. Experimental systems were maintained at 27.5–28.7 °C, 31.0–33.8 ppt salinity. Ammonia, nitrite, and nitrate were maintained at ≤0.25, ≤0.25, and ≤80.00 ppm, respectively. Animal care and procedures used in this trial were approved by Roger Williams University Animal Care and Use Committee (IACUC protocol R-13-12-20).

Wet weight (g) and standard length (mm) was determined for each fish on day 0 and 41 (*N* = 120 and 112, respectively). Fish were individually collected using a dip net and blotted dry with a towel prior to measurements. Wet weight was measured by placing each fish on the center of a digital balance, and standard length per fish was measured from photographs of each individual using ImageJ digital imaging software (Schneider, Rasband & Eliceiri, 2012). Specific growth rate (SGR as g ^−1^) was calculated as ((ln*W*_*f*_ − ln*W*_*i*_ × 100)∕*t*) where ln*W*_*f*_ = the natural logarithm of the final weight, ln*W*_*i*_ = the natural logarithm of the initial weight, and *t* = time (days) between the two measures.

On day 41, three fish were randomly selected from each treatment (*N* = 12 total), freeze-dried for 48 hr, homogenized using a mortar and pestle, and stored in borosilicate vials. Processed samples were then shipped to the New Jersey Feed Laboratory for whole body proximate, amino acid, and fatty acid analyses (% composition).

The foregut of three additional fish from each treatment (*N* = 12 total) was removed via dissection and immediately frozen on dry ice for gut microbial DNA analysis. DNA was extracted from the entire foregut of each animal using MoBio PowerSoil^®^ DNA Isolation Kits (Carlsbad, CA, USA). DNA was amplified using nested PCR, with initial amplification using primers 27F and 1525R that targeted the V1–V6 section of the 16S rRNA gene (Coates et al., 1999). A second amplification using uniquely barcoded primers 515F and 808R (Caporaso et al., 2012) were used to generate final amplicons for sequencing. The amplicons were gel purified using the Qiagen QIAquick^®^ Gel Extraction Kit following the manufacturer’s instructions (Valencia, CA, USA), and then were sequenced using an Illumina MiSeq high throughput DNA sequencer. The resulting sequences were quality filtered and analyzed in QIIME using default parameters (Caporaso et al., 2010).

### Digestibility of KBM using Atlantic salmon

Atlantic salmon (*Salmo salar*; *N* = 96; 635 ± 97 g wet wt) were stocked at 16 fish/tank into six 417 L fiberglass tanks (0.265 m^3^) comprising a 7,500 L flow-through recirculating system with a drum filter, bio-filter, and 1200 L sump. Brackish well water (∼2 ppt salinity) was supplied to the system (19 L min^−1^) and water quality was monitored weekly to ensure that a healthy environment was maintained during the trial. Dissolved oxygen (90–125%) and temperature (11.5–12.2 °C) were monitored daily. Animal care and procedures used in this trial were approved per USDA-ARS Animal Care and Use Committee (IACUC FY2014-001).

To determine the digestibility of KBM, a reference diet was formulated and then KBM was added in a standard 70%/30% (Table 3). Each of the six experimental tanks was randomly assigned to one experimental treatment, totaling three replicates per treatment. All tanks were fed the control diet (C; Table 3) for 7-days prior to initiating the experimental treatments. The basal diet contained 40% protein and 25% lipid with an estimated digestible energy of 19.6 kJ g^−1^. Fish were fed three times/day; at 800 and 1200 hours using automatic feeders (Arvo-Tec Oy, Huutokoski, Finland) and at 1600 hours by hand to apparent satiation. The feeding software was developed from experimental growth models validated from commercial data and different genetic stocks (From & Rasmussen, 1984; Ruohonen & Mäkinen, 1992; Ursin, 1967).

Fecal material was collected from each tank 18 hr post feeding on day 2 and day 4 by manual stripping (Austreng, 1978; Hajen et al., 1993). All fish in each tank were sedated with tricaine methylsulfonate (MS 222, 0.1 g L^−1^) and physically restrained. Pressure was applied to the abdomen to initiate defecation into clean stainless steel pans. Fish were returned to their respective tanks and allowed to recover from handling. Fecal samples collected from all fish (*N* = 6) in each tank were pooled as one composite sample/tank, averaging ≥5 g of dried feces. The fecal material was dried at 60 °C for 24 hr, placed into plastic bags and stored at −20 °C until analysis.

The methods of Cho, Slinger & Bayley (1982); Cho, Slinger & Bayley (1982)) and Bureau, Harris & Cho (1999) were used to estimate apparent digestibility coefficients. Yttrium oxide served as the inert maker.

The diets and fecal material were analyzed for organic matter by drying the samples at 120 °C for 2 hr and ashing the dried samples at 550 °C for 3 hr (AOAC, 1990). Organic matter was calculated as 100 minus ash content. The diets and fecal material were analyzed for lipid content by ether extraction using an Ankom lipid extraction instrument (XT-10; Ankom Technologies, Macedon, NY, USA). Crude protein was determined by the Dumas method (Ebeling, 1968) using a Leco Nitrogen Determinator (FP 528; Leco Corporation, St. Joseph, MI, USA). Energy was determined by using a Parr Instruments calorimeter (Model 1281; Moline, IL, USA). Yttrium oxide determination was by inductively coupled plasma atomic emission spectroscopy (University of Idaho Analytical Laboratory, Moscow, ID, USA). Amino acid analysis was performed using a Beckman 7300 Amino Acid Analyzer (University of Missouri Analytical Lab, Columbia, MO, USA).

Apparent digestibility coefficients of each nutrient in the experimental diets were calculated according to the following equations (Kleiber, 1961; Forster, 1999):

\begin{eqnarray*}& & {\mathrm{ADCN}}_{\mathrm{diet}}=100-100\{\text{%}\mathrm{Y d}\;\mathrm{X}\;\mathrm{Nf}\}/\{\text{%}\mathrm{Y f}\;\mathrm{in\;feces\;X\;Nd}\} \end{eqnarray*}

\begin{eqnarray*}& & {\mathrm{ADCN}}_{\mathrm{ingredient}}=\{(a+b)\mathrm{X}\;{\mathrm{ADCN}}_{\mathrm{t}}-(a\;\mathrm{X}\;{\mathrm{ADCN}}_{\mathrm{r}})\}{b}^{-1} \end{eqnarray*}

where, ADCN = apparent digestibility coefficient of nutrient; Yd = % Yttrium oxide in diet; Nf = % nutrient in feces; Yf = % Yttrium oxide in feces; Nd = % nutrient in diet; *p* = proportion of test ingredient in the test diet; *a* = (1 − *p*) × nutrient content of the reference diet; *b* = *p* × nutrient content of the test ingredient; *t* = apparent digestibility coefficients of the nutrient in the test diets; *r* = apparent digestibility coefficients of the nutrient in the reference diet.

### Statistical analyses

Statistical analyses were conducted using JMP 8.0, SAS, Inc. (Cary, NC, USA). The effect of experimental diets on shrimp growth rate and feed conversion ratio, and the effect of experimental diets on animal growth rate, was assessed using a one-factor analytical design. Normal data (indicated by a Jarque-Barre (JB) test for normality; Zaiontz, 2015) were tested with a one-way analysis of variance (ANOVA). If the JB test indicated non-normal data, the analyses were conducted nonparametrically using a Kruskal-Wallis test. Post-hoc comparisons of normal data were made and *post hoc* comparisons of orthogonal contrasts from ANOVA tests were examined using the Real Statistics Resource Pack software (Release 4.9, Zaiontz, 2015). The effect of experimental diet on shrimp taste was determined using a two-factor (treatment and order of preference) chi-square goodness-of-fit test. The effect of experimental diets on grunt microbiome was determined using adonis, a permutational multivariate analysis of variance, (PERMANOVA) implemented in R, and the similarities among treatments were calculated using the Bray-Curtis similarity metric and were visualized using a principal coordinates analysis in QIIME (Caporaso et al., 2012). The significance level for all analyses was set at *P* ≤ 0.05. All values are presented as mean ± standard error.

## Results

### Pacific white shrimp

Diet had no effect on shrimp survival (one-way ANOVA, *F*_2,9_ = 2.4, *p* > 0.1, combined average =84.7 ± 5.6%); however, diet did influence shrimp growth (one-way ANOVA, % weight gain, *F*_2,9_ = 5.4, *p* < 0.05; SGR, *F*_2,9_ = 8.6 g d^−1^, *p* < 0.01). Shrimp fed diet with 100% FM replacement (SHR-KH) grew less than those fed the control diet (SHR-C), and shrimp fed diet with 50% FM replacement (SHR-KL) showed growth intermediate to, and not statistically different from either SHR-C or SHR-KH (Table 4). Diet influenced shrimp feed efficiency (one-way ANOVA, *F*_2,9_ = 5.27, *p* < 0.05, Table 4). The food conversion ratio (FCR) of shrimp fed diets containing KBM (SHR-KL and SHR-KH) were not statistically different than those fed the control diet (SHR-C, 1.70 ± 0.12, orthogonal contrast, Q Test = − 0.99, *p* > 0.7). The shrimp fed the SHR-KH had a statistically greater FCR than those fed SHR-KL (orthogonal contrast, Q Test = − 4.48, *p* < 0.05).

**Table 4:** Shrimp growth. The growth expressed as percent weight gain and the specific growth rate (SGR, g d^−1^) of shrimp fed one of three experimental diets. Diets indicate the amount of fish meal substituted by KBM, with C indicating a control with no substitution, KL with 50% of fish meal substituted by KBM, and KH with a 100% substitution. Superscripts indicate statistical similarity (one-way ANOVA, Tukey’s HSD, *p* > 0.5).

	% Weight gain	SGR g ^−1^	FCR
SHR-C	150.9 ±4.9%^a^	2.92 ±0.05^a^	1.70 ±0.12^a,b^
SHR-KL	140.8 ±10.9%^a,b^	2.81 ±0.09^a,b^	1.59 ±0.06^a^
SHR-KH	128.6 ±11.8%^b^	2.64 ±0.14^b^	1.95 ±0.05^b^

In the consumer taste trial, diet did influence shrimp preference (*df* = 4, *χ*^2^ = 9.8, *p* < 0.05). This was largely because shrimp fed diet SHR-C received more votes as “most preferred” (50% of all first place votes). The shrimp fed diets SHR-KL and SHR-KH each received 25% of the “most preferred” votes. However, there was no diet difference in the shrimp that was voted “least favorite”; each diet received 1/3 of the last place votes.

### Smallmouth grunt

Diet had no effect on grunt mortality, length, weight, condition factor, or specific growth rate (SGR) (for all variables, one-way ANOVA *F*_3,8_ > 2.85). Only 12 of the 120 grunts died during the experiment, and no more than one fish was lost per tank. For all fish, the average weight increase was 353.2 ± 45.9%, length increase was 48.4 ± 6.2%, condition factor was 2.3 ± 0.2, and SGR was 3.8 ± 0.3 g d^−1^. Average feed conversion ratio (FCR) per treatment ranged from 1.09–1.24. Diet did affect fish proximate composition (one-way ANOVA *F*_3,8_ = 4.3, *p* < 0.05), where fish fed the control diet (GRU-C1) had the greatest protein content (dry matter, 56.3 ± 1.9%), while those fed diet with 50% FM replacement (GRU-KH) had the lowest (52.93 ± 1.2), while the control diet with pigment (GRU-C2) and the diet with 10% FM replacement (GRU-KL) were intermediate and not statistically different from the extremes (R30: 53.6 ± 1.0%; C+: 53.76 ± 1.1%. The percent fat on a dry matter basis, while not significant, exhibited the opposite trend to protein with the fish fed GRU-C having 22.9 ± 2.2% fat, while those fed diet GRU-KL had 27.3 ± 1.3% fat.

**Figure 1:**
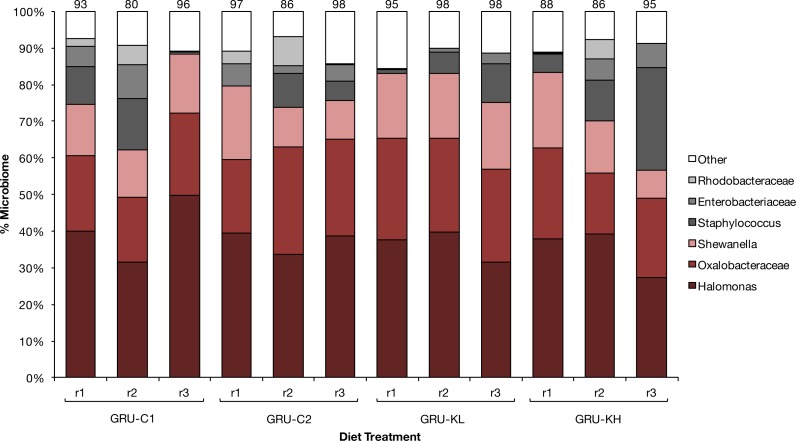
Microbiome. Core gut microbial composition of 12 smallmouth grunt (*H. chrysargyreum*) exposed to four experimental feeds (*N* = 3 per feed) used to test KnipBio single cell protein (KnipBio meal; KBM) as a fishmeal substitute, where GRU-C1m **GRU**nt **C**ontrol feed (modelled after Alam et al., 2012; Alam, Watanabe & Carroll, 2008; Alam et al., 2009), GRU-C2, GRU-C1 with 80 ppm carotenoid addition, and GRU-KL and GRU-KH are control feed with fishmeal replaced with KBM; KL, **K**nipBio meal **L**ow (10% replacement) and KH, **K**nipBio meal **H**igh (50% replacement). Numbers above bars indicate the proportion of the microbiome represented by the six core groups (*N* = 6).

Diet did not significantly affect grunt gut microbial community (adonis, *p* > 0.05). Regardless of diet, ∼60% of the fish gut microbiome was composed of three types of bacteria: *Halomonas*, *Oxalobacteracaea*, and *Shewanella*. The composition of the remaining microbial community varied among individuals, but differences in the total number of operational taxonomic units among treatments was not statistically significant (Fig. 1), and there was no clear treatment grouping on the principal coordinates analysis.

### Atlantic salmon

For salmon, KBM inclusion had no measurable effect on the Apparent Digestibility Coefficient (ADC) value. The ADC for protein was slightly greater for the animals fed the control diet compared to the 30% inclusion KBM diet (67.8 ± 2.8 and 63.0 ± 3.1 (averages ±1 S.D.) respectively). Diet did have a positive although insignificant influence amino acid digestibility, as the KBM diet (SAL-K) resulted in better digestibility for seven of the eight essential amino acids and six of the 11 non-essential amino acids (Table 5) than the control (SAL-C) diet (for all amino acids, binomial probability, ∏ = 0.5*p* < 0.08).

**Table 5:** Protein digestibility. Protein and amino acid digestibilities (%) of Atlantic salmon (*S. salmar*) exposed to two experimental feeds used to determine the viability of KnipBio single cell protein (KnipBio Meal; KBM) as a fishmeal substitute, where **SAL-C**, **SAL**mon **C**ontrol diet (*modelled after* Gaylord et al., 2009) and **SAL-K**, **SAL**mon control diet + 297.0 g kg^−1^ **K**BM.

Nutrient	Digestibility (%)	
	SAL-C	SAL-K
*Crude Protein*	72.32	69.12
*Essential amino acids*		
Lysine	74.76	79.37
Methionine	75.46	82.01
Histidine	78.14	80.95
Isoleucine	70.72	77.72
Leucine	70.51	81.63
Phenylalanine	75.83	75.26
Tryptophan	77.46	77.71
Valine	74.65	78.82
*Nonessential amino acids*		
Aspartic acid	69.13	70.24
Threonine	68.52	71.37
Serine	79.19	79.89
Glutamine	80.53	79.54
Proline	74.62	80.25
Glycine	70.26	69.73
Alanine	70.35	75.54
Cysteine	60.99	69.56
Tyrosine	77.94	75.80
Arginine	90.03	83.42

## Discussion

Ultimately, innovative sources of protein are required to meet the feed needs of aquaculture (Naylor et al., 2009) if aquaculture will meet its projected doubling by 2030 (Kobayashi et al., 2015). The production of single cell proteins (SCP) is one potential high quality protein alternative to fishmeal, and has the potential to stabilize the rising aquafeed input costs and address the over-harvesting of pelagic fisheries for use in fishmeal. This will ultimately lead to a more resilient and sustainable global food supply.

Here we demonstrated the broad applicability of KnipBio Meal, made from *Methylobacterium extorquens*, as a viable protein source for use in aquafeeds. When fed to fishes it resulted in equivalent performance in growth for grunts, and ADC for salmon as trials using traditionally formulated diets containing fishmeal. The salmon demonstrated higher digestibility for amino acids in the KBM diets. The FCR of shrimp was best when there was 50% substitution with KBM, yet growth (weight gain and SGR) was greatest in the control diets. Overall, this suite of results is encouraging given that there was no engineering, selection, or tuning of the bacteria to be more suitable as an aquaculture feed or to achieve advanced feed formulation considerations. These results also suggest that KBM contains no anti-nutritional properties, a common hurdle to overcome in the adoption of plant-derived alternate feed ingredients (Francis, Makkar & Becker, 2001; Refstie et al., 2005). The anti-nutritional properties may be a reason that prior studies on plant-based feed substitution have reported changes in the intestinal microbiome, with associated decreased health outcomes (Desai et al., 2012; Green, Smullen & Barnes, 2013; Ingerslev et al., 2014; Rhodes, Johnson & Myers, 2016). This diet-based gut microbiome modulation (Estruch et al., 2015) can help indicate inadequate diets. Thus, our observed lack of treatment related difference in the gut microbiome of the smallmouth grunts in this study should be considered significant in that it suggests *M. extorquens* is of sufficient quality in this early stage testing to garner support as a SCP feed additive.

Diet apparent digestibility coefficient (ADC) values for protein were slightly lower for the KBM diet compared to fishmeal (73.2%–69.1%), a result consistent with prior findings (Skrede et al., 1998; Storebakken et al., 2004). Amino acids can drive ADC values, and the relative proportion of lysine, methionine and histidine could influence the greater ADC in the fishmeal.

While the initial tests with KBM in aquaculture feed are promising, further development is still required, particularly in two areas. First, a diet treatment difference was observed that resulted in a minor difference in shrimp taste. Often, studies involving protein substitution in aquaculture will result in a less palatable product (De Francesco et al., 2004). Within this study, while the shrimp fed the control diets were the most preferred, it is noted that all treatments were represented equally within the third-ranked (least-preferred) category. Thus, none of the treatments had an unpalatable flavor or texture, and, in the words of Tamar Haspel, writer for The Plate, the shrimp fed the KBM diets “tasted like shrimp” (Haspel, 2015). A second issue associated with sub-scale pellet manufacturing was the inclusion of air bubbles in the shrimp diet SHR-KH (100% KBM replacement) that did not sink as well as the other two diets (SHR-LK and SHR-C). While all pellets were consumed by shrimps, those fed SHR-HK did need to swim in the water column to retrieve some of the pellets, while those fed SHR-LK and SHR-C mostly fed off the tank floor. Additionally, diet SHR-KH seemed to be less palatable as the shrimps had a lower feed efficiency of this than the other diets. Whether this is an absence of an attractant not replaced in SHR-KH or the absence of a critical nutritional component like methionine (DA Davis, pers. comm., 2015) in the diet, the exact cause is unknown at this time. While minor adjustments to the diet formulation will be necessary, they likely will not be onerous, and this work gives promise for *M. extorquens* as a SCP in aquafeeds.

In early stage technology development, sub-optimal bioprocessing volumes, feed pellet manufacturing and other related operation scales can magnify negative results. As such, the cost effectiveness of KBM production has not yet been validated and was beyond the scope of this study. However, one immense potential efficiency advantage of SCP as a protein source is to small amount of space required for production. An estimated 40.5 ha SCP facility can match the protein production of a 4047 ha soy operation, dramatically reducing the environmental footprint of production. The ability to make a fishmeal alternative, combined with vital ingredients like anti-oxidant carotenoids, both simplifies and diversifies the raw ingredients available to feed manufacturers while avoiding the exploitation of marine resources. Continued work to scale up KBM and other SCP solutions is required to create a cost effective near-term solution for alternate protein sources for aquafeeds. Based on this research, we believe KBM can be used as a dietary component, and upon further investigation, could serve as a complete fishmeal substitution for aquafeeds without compromising feed performance. The use of this SCP will ensure future food security by creating novel resources to grow larger volumes of aquatic protein that does not complete with humans and terrestrial livestock for limited resources.

##  Supplemental Information

10.7717/peerj.3170/supp-1 Data S1 KBM raw data

## Additional Information and Declarations

### Data Availability

The following information was supplied regarding data availability: The raw data has been supplied as Data S1.
